# Global Jitter Motion of the Retinal Image Dynamically Alters the Receptive Field Properties of Retinal Ganglion Cells

**DOI:** 10.3389/fnins.2019.00979

**Published:** 2019-09-13

**Authors:** Akihiro Matsumoto, Masao Tachibana

**Affiliations:** ^1^Department of Psychology, Graduate School of Humanities and Sociology, The University of Tokyo, Tokyo, Japan; ^2^Ritsumeikan Global Innovation Research Organization (R-GIRO), Ritsumeikan University, Kusatsu, Japan; ^3^Danish Research Institute of Translational Neuroscience (DANDRITE), Department of Biomedicine, Aarhus University, Aarhus, Denmark; ^4^Research Organization of Science and Technology, Ritsumeikan University, Kusatsu, Japan

**Keywords:** retina, retinal ganglion cells, receptive field, eye movements, gap junctions

## Abstract

Fixational eye movements induce aperiodic motion of the retinal image. However, it is not yet fully understood how fixational eye movements affect retinal information processing. Here we show that global jitter motion, simulating the image motion during fixation, alters the spatiotemporal receptive field properties of retinal ganglion cells. Using multi-electrode and whole-cell recording techniques, we investigated light-evoked responses from ganglion cells in the isolated goldfish retina. Ganglion cells were classified into six groups based on the filtering property of light stimulus, the membrane properties, and the cell morphology. The spatiotemporal receptive field profiles of retinal ganglion cells were estimated by the reverse correlation method, where the dense noise stimulus was applied on the dark or random-dot background. We found that the jitter motion of the random-dot background elongated the receptive filed along the rostral-caudal axis and temporally sensitized in a specific group of ganglion cells: Fast-transient ganglion cells. At the newly emerged regions of the receptive field local light stimulation evoked excitatory postsynaptic currents with large amplitude and fast kinetics without changing the properties of inhibitory postsynaptic currents. Pharmacological experiments suggested two presynaptic mechanisms underlying the receptive field alteration: (i) electrical coupling between bipolar cells, which expands the receptive field in all directions; (ii) GABAergic presynaptic inhibition from amacrine cells, which reduces the dorsal and ventral regions of the expanded receptive field, resulting in elongation along the rostral-caudal axis. Our study demonstrates that the receptive field of Fast-transient ganglion cells is not static but dynamically altered depending on the visual inputs. The receptive field elongation during fixational eye movements may contribute to prompt firing to a target in the succeeding saccade.

## Introduction

In living animals, the retina receives unstable visual inputs induced by movements of body, head, and eyes (Land, 2009). Even when an animal is fixating an object, the whole image on the retina is shifted by the presence of incessant microscopic eye movements (“fixational eye movements”) (Martinez-Conde et al., 2004; Rucci and Poletti, 2015). Once fixational eye movements are stabilized, visual perception fades rapidly (Yarbus, 1967; Murakami, 2006). It has been shown that fixational eye movements affect visual performance such as visual acuity (Keesey, 1960), contrast sensitivity (Tulunay-Keesey and Jones, 1976), and detection of visual features (Rucci et al., 2007). Physiological evidence indicates that the image motion induced by fixational eye movements prevents the adaptation of neural activity (Martinez-Conde et al., 2004; Rucci and Poletti, 2015). Involuntary small eye movements during fixation, called microsaccades, increase firing activity of primate visual cortical neurons (V1, Martinez-Conde et al., 2000; area MT, Bair and O’Keefe, 1998). Retinal ganglion cells (GCs) respond with higher firing rate to the jittering image than the static image, together with synchronization (Greschner et al., 2002) or with decorrelation (Segal et al., 2015).

In natural vision, animals repeat fixations and brief gaze shifts (saccades) (Wurtz, 2008; Land, 2009). It has been shown that firing of GCs is increased (Noda and Adey, 1974) or suppressed (Roska and Werblin, 2003; Tien et al., 2015) by saccade-like image shift depending on cell types. A burst firing is evoked by image recurrence across eye-movement-like image transitions in mouse specific GCs (Krishnamoorthy et al., 2017). Glycinergic and GABAergic inhibitory inputs from amacrine cells seem to contribute to the firing modulation during the rapid image shift (Roska and Werblin, 2003; Tien et al., 2015; Krishnamoorthy et al., 2017).

In our previous study, applying a multi-electrode array to GCs in the goldfish isolated retina, we showed that firing properties of specific GC groups were modulated by a rapid shift of a target following a period of jitter motion of a global random-dot background (Matsumoto and Tachibana, 2017). In particular, the response latency to a rapidly moving target was shortened in Fast-transient (Ft) GCs only when rapid motion was preceded by global jitter motion. Intriguingly, the response modulation was specific to rapid motion along the rostral-caudal axis. These results suggest that the receptive field (RF) properties of Ft GCs may have been altered during a period of global jitter motion prior to rapid motion. However, global jitter motion *per se* did not evoke firing in Ft GCs, and thus, it remains to be solved how global jitter motion alters the RF properties of Ft GCs and what mechanisms underlie the alteration.

Here, applying the whole-cell clamp technique as well as the multi-electrode technique to goldfish retinal GCs, we analyzed the effects of global jitter motion on the spatiotemporal RF profiles. We found that the RF of Ft GCs was spatially elongated along the rostral-caudal axis and temporally sensitized by jitter motion. At the newly emerged regions, local light stimulation frequently evoked excitatory postsynaptic currents with large amplitude and fast kinetics. Pharmacological experiments suggested that the RF alterations were mediated by activation of electrical coupling between bipolar cells and GABAergic inhibition from amacrine cells to bipolar cell terminals. Elongation of the RF of Ft GCs during jitter motion may contribute to prompt response to a rapidly moving target in the succeeding saccade.

## Materials and Methods

### Experimental Model and Subject Details

Goldfish (*Carassius auratus*; 8–12 cm; *n* = 62) was used for the experiments. Animals were kept in a room maintained at 23°C on a 12 h light/dark cycle. All protocols complied with “A Manual for the Conduct of Animal Experiments in The University of Tokyo” and “Guiding Principles for the Care and Use of Animals in the Field of Physiological Sciences, The Physiological Society of Japan.”

### Method Details

#### Retinal Preparation

Goldfish were dark-adapted for more than 1 h before experiments. Under a dim red light, a goldfish was double-pithed, and eyes were enucleated. The following procedure was performed under a stereomicroscope equipped with infrared (IR) image converter (C5100, Hamamatsu photonics) and IR illuminator (HVL-IRM, Sony). After the cornea and lens were ablated, the eye cup was treated with a mixture of hyaluronidase and collagenase (4 mg/mL each, Sigma-Aldrich Corp.) for a few min. A small cut was made at the dorsal part of the eye cup as a landmark and thus the ventral retina isolated from the pigment epithelium was properly oriented and positioned on the multi-electrode array or in the recording chamber for whole-cell recordings.

#### Recordings

**FIGURE 1:**
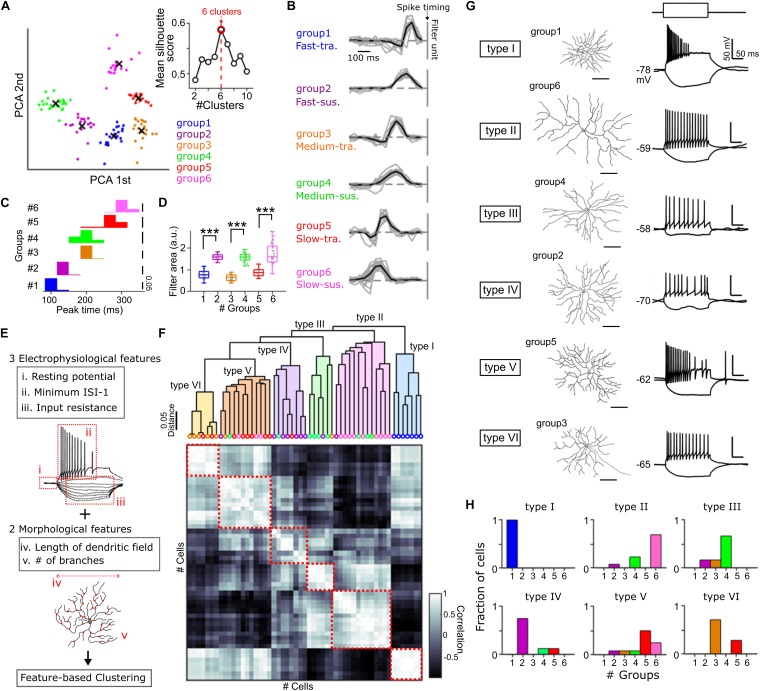
Cell type classification based on the physiological and anatomical features. **(A)** Principal component analysis (PCA) of the temporal RF profile. x, centroid of each cluster. Inset, relationship between mean silhouette score and the number of clusters in k-means clustering. Red, the estimated optimal number of clusters. **(B)** The temporal RF profile of each GC group. Black vertical line, spike timing. Gray, individual cells. Black, mean. 20 Fast-transient (Ft), 18 Fast-sustained (Fs), 22 Medium-transient (Mt), 22 Medium-sustained (Ms), 18 Slow-transient (St), 27 Slow-sustained (Ss) GCs. **(C,D)** Histograms of the peak time panel **(C)** and the area of temporal RF profile panel **(D)** in each group. Gray dots, individual cells. **(E)** The physiological and morphological features used for a hierarchical clustering panel **(F)**. **(F)** Top, dendrogram based on the hierarchical clustering. Colored circles, identity of GC groups based on the filtering property panel **(A)** of individual cells. Bottom, a matrix of correlation of five features between individual cells. Red square, correlation within identified six types (type I –VI). 53 GCs. **(G)** Examples of cell morphology (left) and membrane potential changes induced by current pulses (right, –50 and +80 pA in amplitudes, 200 ms in duration) for each type panel **(F)**. **(H)** Fraction of GC groups included in each type. ^∗∗∗^*p* < 0.001. See also Supplementary Figure S1.

**FIGURE 2:**
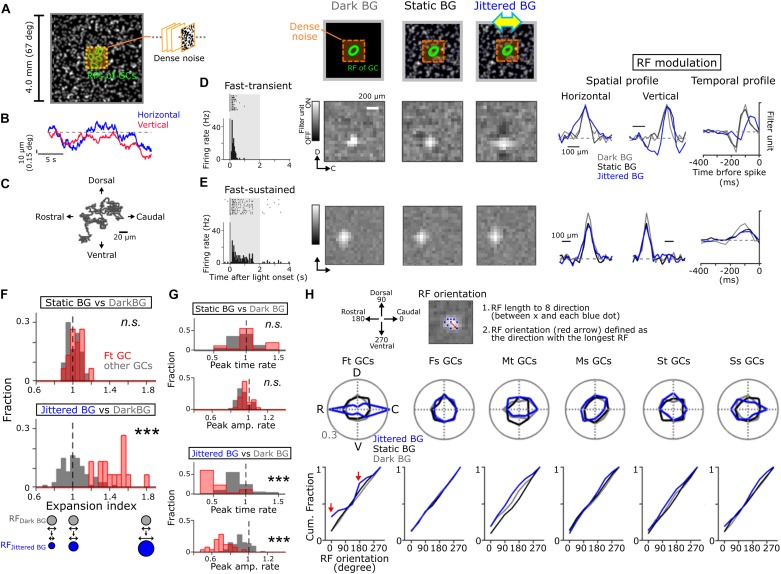
Global jitter motion alters the RF profiles of Fast-transient GCs. **(A)** A schematic of stimulus. Dense noise stimulus (orange, 1.6 × 1.6 or 0.96 × 0.96 mm) was presented on the random-dot background (4 × 4 mm, mean contrast; 45.6%, mean luminance; 6.67 cd/m^2^). The random-dot background was moved in four cardinal directions randomly (4 μm/50 ms; Jittered BG). **(B)** Examples of random-walk trajectory in the horizontal (blue) and vertical (red) directions. **(C)** 2D plots of the jitter motion trajectory (20 s). **(D,E)** Top, schematics of stimuli. Left, firing to a flash (gray band; size, 1 × 1 mm; duration, 2 s; 50% contrast increment) in Fast-transient (Ft) **(D)** and Fast-sustained (Fs) **(E)** GCs. Middle, the spatial RF profile under the Dark (gray), Static (black), and Jittered (blue) BG conditions. Right, the spatial and temporal RF profiles (upper, Ft GC; lower, Fs GC). **(F,G)** Expansion index panel **(F)** and temporal RF profile (**G**; upper, peak time; lower, peak amplitude): top, Static BG vs. Dark BG; bottom, Jittered BG vs. Dark BG. Fraction was calculated separately for Ft GCs (red, *n* = 17) and other GCs (gray, *n* = 73). **(H)** Top, a schematic for calculation of the RF orientation. The RF orientation was determined as a direction (red arrow) with the longest RF length (between × and blue dots). Middle; polar plot of the RF orientation under the Dark (gray), Static (black), and Jittered (blue) BG conditions for each GC group: 17 Ft GCs, 16 Fs GCs, 10 Medium-transient (Mt) GCs, 14 Medium-sustained (Ms) GCs, 14 Slow-transient (St) GCs, and 19 Slow-sustained (Ss) GCs. Bottom; cumulative fraction of the RF orientation. Red arrows; cumulative fraction at the horizontal axis (0 and 180 degree) under the Jittered BG condition. ^∗∗∗^*p* < 0.001. See also Supplementary Figure S1 for physiological and morphological features of each GC group, and Supplementary Figure S2 for visual stimulation.

For multi-electrode recordings (Figures 1, 2), the isolated retina was placed on the electrode array (60 electrodes, electrode diameter 30 μm, electrode spacing 200 μm; 60pMEA200/30iR-Ti, Multichannel Systems) with the GC layer facing down, and light stimulation was applied from the photoreceptor side. The recorded signals were stored at 16 kHz through AD converter with 16 channels (PowerLab 16/35; AD Instruments). The retina was continuously superfused with an extracellular solution bubbled with 95% O_2_/5% CO_2_ at the rate of 1 mL/min. The solution consisted of (in mM) 106 NaCl, 2.6 KCl, 28 NaHCO_3_, 2.5 CaCl_2_, 1 MgCl_2_, 1 Na-pyruvate, 10 D-glucose, 4 mg/L phenol red. For pharmacological experiments, drugs were added to the extracellular solution. Drugs were obtained from Sigma-Aldrich. Recorded spike discharges were band-pass filtered between 100 and 3,000 Hz and sorted into single unit activities by principal component analysis (PCA) and the template-matching method with custom programs using MATLAB (Lewicki, 1998; Zhang et al., 2004; Matsumoto and Tachibana, 2017). For further analysis, we selected up to 3 single units/electrode, showing robust light-evoked responses based on two criteria: responsibility and reliability. Responsibility was evaluated by comparing mean firing probability before and during light stimulus using two-tailed *t*-test. Reliability was evaluated by trial-to-trial variability in spike counts during light stimulus based on Pearson’s correlation. To verify the accuracy of sorting, we calculated the auto-correlation of the sorted spike train for each unit, and confirmed a lack of events corresponding to the refractory period of spikes (Supplementary Figure S1A) (Lewicki, 1998; Rey et al., 2015).

**FIGURE 3:**
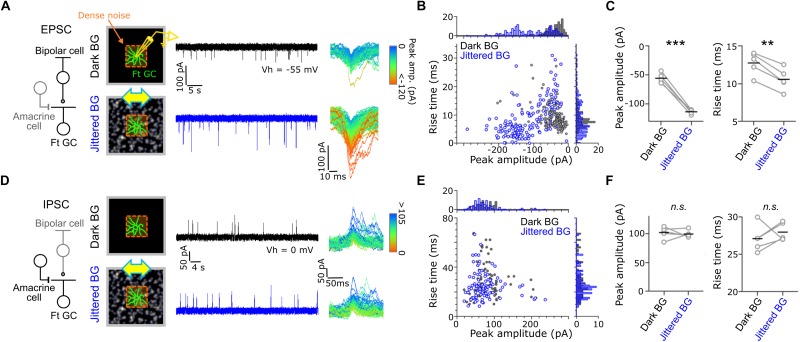
Synaptic inputs to Fast-transient GCs. **(A)** Excitatory postsynaptic currents (EPSCs) evoked by dense noise stimulation under the Dark (upper, black) and Jittered (lower, blue) BG conditions. Left, a schematic of synaptic inputs to a Ft GC. Middle, a schematic of recordings and evoked EPSCs in a Ft GC voltage-clamped at –55 mV (V_h_). Right; superimposed excitatory events, in which each color indicates different peak amplitude. **(B)** Plot of peak amplitude and rise time for each excitatory event under the Dark (gray dots) and Jittered (blue dots) BG conditions. Data from the Ft GC shown in panel **(A)**. **(C)** Mean peak amplitude (left) and rise time (right) in individual cells (circle) under the Dark and Jittered BG conditions. Black line, mean. 5 Ft GCs. **(D–F)** Inhibitory postsynaptic currents (IPSCs) recorded from Ft GCs voltage-clamped at 0 mV. 5 Ft GCs. ^∗∗^*p* < 0.01; ^∗∗∗^*p* < 0.001. See also Supplementary Figure S3.

**FIGURE 4:**
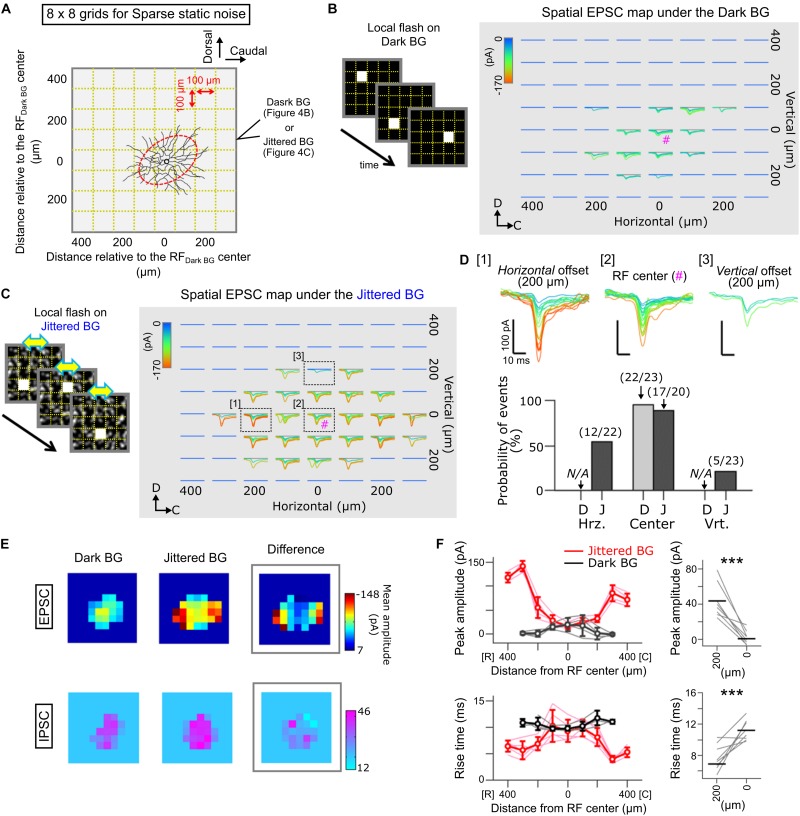
Spatial map of EPSCs evoked by local flash stimulation in Ft GCs. **(A)** A schematic of sparse noise stimulation on the Dark **(B)** and Jittered BG **(C)**. The sparse noise was a sequence of a static flash (size, 100 × 100 μm; duration, 50 ms) randomly applied at one of the 8 × 8 grids (gold dotted lines). Stimulus location was described from the RF center estimated under the Dark BG condition (red). **(B,C)** Superimposed EPSCs evoked by local flash stimulation presented on the Dark **(B)** and Jittered **(C)** BGs. EPSCs are colored based on the peak amplitude. #; the RF center. **(D)** Top, EPSCs evoked at three locations: [1] horizontally 200 μm away from the RF center, [2] the RF center, and [3] vertically 200 μm away from the RF center. Bottom, probability of EPSC events evoked by local flash stimulation. Numbers, EPSC events/stimulation. N/A, no evoked EPSCs. D, Dark BG. J, Jittered BG. **(E)** Heatmaps showing the peak amplitude of EPSCs (top) and IPSCs (bottom) evoked by local flash stimulation on the Dark (left) and Jittered (center) BGs. Right, a heatmap showing differences of amplitude between the Dark and Jittered BGs. Data from the Ft GC **(C)**. **(F)** Left, peak amplitude (upper) and rise time (lower) of the evoked EPSCs are plotted against the position relative to the RF center (5 Ft GCs) under the Dark (black) and Jittered (red) BG conditions. Thin lines, data from individual cells. Right, mean peak amplitude (upper) and rise time (lower) of EPSCs evoked by flash stimulation 200 μm away from the RF center and at the RF center (0 μm). ^∗∗∗^*p* < 0.001.

**FIGURE 5:**
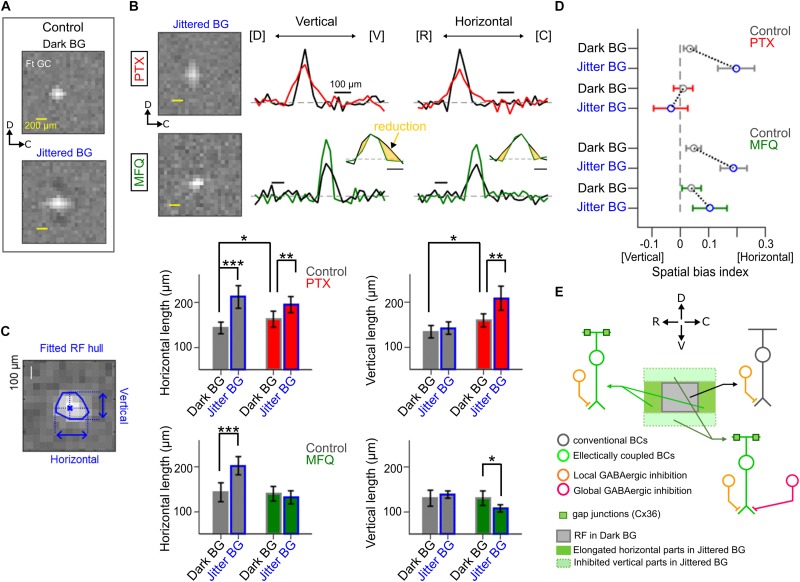
Effects of pharmacological blockade of GABAergic inhibition and electrical coupling. **(A)** The spatial RF profile of a Ft GC under the Dark and Jittered BG conditions in control solution. **(B)** The spatial RF profile in the presence of picrotoxin (PTX, 100 μM, red) and mefloquine (MFQ, 10 μM, green). Left, the estimated RF under the Jittered BG condition. Right, the spatial RF profile along the vertical (dorsal–ventral) and horizontal (rostral–caudal) axes in control (black), PTX (red), and MFQ (green) solutions. **(C)** Left, a schematic for calculation of the horizontal and vertical lengths of the RF (blue). Right, horizontal and vertical lengths of the RF under the Jittered (blue) and Dark (gray) BG conditions in control (gray), PTX (red) and MFQ (green) solutions. 7 Ft GCs. **(D)** Spatial bias index (see section “Method Details”) under the Jittered (blue) and Dark (gray) BG conditions in control (gray), PTX (red), and MFQ (green) solutions. Circle and error bars, mean ± SD. 7 Ft GCs. **(E)** A schematic model to explain the horizontal RF elongation. A Ft GC receives excitatory inputs from distinct BC populations: conventional BCs (dark gray) which evoke small and slow EPSCs, and electrically coupled BCs (right green) which evoke large and fast EPSCs under the Jittered BG condition. BCs receive GABAergic inhibition (orange) from local ACs which are activated by local stimulation under the Dark BG condition. The electrically coupled BCs at the dorsal and ventral sides additionally receive global GABAergic inhibition (magenta) from wide-field (or electrically coupled) ACs which are activated by the Jittered BG. ^∗^*p* < 0.05; ^∗∗^*p* < 0.01; ^∗∗∗^*p* < 0.001.

For whole-cell recordings (Figures 1, 3, 4), the isolated retina was placed on the recording chamber with the GC layer facing up, and light stimulation was applied from the photoreceptor side. The retina was continuously superfused with the bubbled extracellular solution. The pipette was filled with intracellular solution (in mM): 128 K gluconate, 10 KCl, 10 Hepes, 0.5 EGTA, 0.05 CaCl_2_, 2 MgCl_2_, 5 ATP-Na_2_, 0.5 GTP-Na_3_, and 0.08% Lucifer yellow-2K (pH 7.4 with KOH) for current-clamp recordings, and 118 CsMeSO_3_, 10 TEA-Cl, 10 Hepes, 0.5 EGTA, 0.05 CaCl_2_, 2 MgCl_2_, 5 ATP-Na_2_, 0.5 GTP-Na_3_, 5 QX314-Br, and 0.08% Lucifer yellow-2K (pH 7.4 with CsOH) for voltage-clamp recordings. E_Cl_ was calculated as −55 mV. The membrane potential was corrected for liquid junction potential which was measured before experiments. For pharmacological experiments (Figure 5), we added picrotoxin (GABA receptor blocker; 100 μM, Sigma) or mefloquine (gap junction blocker; 10 μM, Sigma) to the extracellular solution. Recordings were performed using EPC 10 (HEKA Electronik) controlled by Pactchmaster (version 2.73.5). Current and voltage records were sampled at 16 kHz and low-pass filtered at 2.9 KHz. We used a borosilicate glass electrode (CNC 1.5; Ken Enterprise), which was pulled by a puller (P97; Sutter Instrument). The resistance of recording pipettes was 6–11 MΩ. For cell group identification, light-evoked spikes were recorded in the cell-attached mode before whole-cell recording.

#### Light Stimulation

Light patterns were generated by Psychtoolbox3 on MATLAB (Mathworks) (Brainard, 1997; Pelli, 1997). For multi-electrode recordings, a multi-electrode array was placed on the stage of an inverted microscope (IX70; Olympus). The light stimulus was projected from a cathode-ray tube display (S501J, refresh rate 60 Hz, 1,280 × 1,024 pixels, Iiyama) to the photoreceptor layer of the retina through optics. For whole-cell recordings, a patch pipette was approached from the ganglion cell side. The light stimulus was projected from a DLP projector (L51W, refresh rate 60 Hz, 1,280 × 1,024 pixels, NEC) to the photoreceptor layer through an objective lens (4 × /0.10, Nikon), with which a condenser lens of an upright microscope (Eclipse E600-FN; Nikon) was replaced. In either recording condition, the stimulus image was not distorted because the electrode was positioned on the opposite side of the incident light.

We used a background frame (4.0 × 4.0 mm) which was either uniformly dark or a Gaussian-filtered (σ, 40 μm) random-dot pattern (51,325 dots/1,000^2^ pixels, 4 μm/pixel, Figure 1A, see details in Matsumoto and Tachibana, 2017). Background Contrast (*C*_BK_) was calculated by

$$C_{\text{BK}}=\left({\text{luminance}}_{\text{BK}}-{\text{luminance}}_{\text{dark}}\right)\,/\,{\text{luminance}}_{\max}$$

where luminance_BK_, luminance_dark_, and luminance_max_ were the mean intensity of the random-dot pattern, 0.11 and 14.4 cd/m^2^, respectively. We introduced global jitter motion to simulate the *in vivo* fixational eye movements of goldfish (Figures 2A–C) (Easter et al., 1974; Mensh et al., 2004). Global jitter motion was a horizontally biased random walk, in which a shift (4 μm/50 ms) to one of the four cardinal direction occurred in each stimulus frame, and the probability of horizontal shift was two times higher than that of vertical shift.

#### Receptive Field Estimation

The spatiotemporal receptive field (RF) was estimated by the reverse correlation method (Meister et al., 1994; Matsumoto and Tachibana, 2017). The retina was stimulated with dense noise consisted of pseudorandom (M-sequence) checkerboard patterns. Each frame (32 × 32 pixels with black or white; pixel size, 50 × 50 μm or 30 × 30 μm) was updated at 30 Hz. The dense noise was placed on the center region (1.6 × 1.6 or 0.96 × 0.96 mm; Figure 2A, orange) of the background (4.0 × 4.0 mm). The checkerboard frames that preceded each spike discharge were averaged (STA, spike-triggered average).

To define the orientation and size of the RF, we determined an “edge” (a position of a pixel with the intensity six times higher than the SD of the intensity in uncorrelated image) along 8 directions (0–315°, *Δ*45°) from the RF “center” (a pixel with maximal intensity). Eight edges were fitted by an ellipse based on the method of least squares. RF size was calculated by a length of major axis in the fitted ellipse. RF orientation (Figure 2H) was defined by the direction with the longest length among eight directions. The temporal RF profile was obtained by calculating the mean intensity of 3 × 3 pixels in the RF center region for a series of the averaged frames.

The temporal RF profile was used for classification of GC groups (Matsumoto and Tachibana, 2017; Figures 1A–D and Supplementary Figure S1B). In brief, we first created an input matrix from the temporal RF profile of each GC, in which the temporal RF profiles were upsampled using *interp* function with a rate of 3 in MATLAB, and smoothed by a moving average filter. After the smoothing, the temporal filters were downsampled to the original rate using *downsample* function in MATLAB. Next, principal component analysis (PCA) was applied to visualize the features of temporal RFs (Figure 1A). Then, clustering was performed by k-means clustering using *kmeans* function in MATLAB. The number of clusters was determined based on the mean silhouette scores [s(*i*)] (Figure 1A),

s⁢(i)=b⁢(i)-a⁢(i)⁢/⁢max⁡{a⁢(i),b⁢(i)},

where *i* is each cell, a(*i*) is average distance between *i* and all other points included in the same cluster, and b(*i*) is the smallest average distance of *i* to all other points (Rousseeuw, 1987; Yuan and Yang, 2019). We adopted the cluster numbers with the highest mean silhouette score for k-means clustering (Martínez et al., 2017).

To quantify the horizontal and vertical lengths of RF, we first fitted a hull to the RF (Figure 5C) using *convex hull* function in MATLAB. A centroid where the horizontal and vertical axes crossed was calculated in the fitted hull, and then, we measured the horizontal and vertical lengths through the centroid. To quantify the spatial bias of the RF profile, the spatial bias index (*SBI*) was defined by,

$$S\,B\,I=\left({\text{length}}_{\text{Horizontal}}-{\text{length}}_{\text{Vertical}}\right)\,/\left({\text{length}}_{\text{Horizontal}}+{\mathrm{length}}_{\text{Vertical}}\right)$$

where length_Horizontal_ and length_Vertical_ are the measured RF length along the horizontal and vertical axes, respectively (Figure 5D). The positive and negative values indicate the horizontal and vertical bias of the RF, respectively.

#### Waveform Analysis

For whole-cell voltage-clamp recordings (Figures 3, 4), wave event was detected as the postsynaptic current when the current amplitude crossed a threshold defined by

$$T\,h\,r\,e\,s\,h\,o\,l\,d=\left|{\text{Mean}}_{\text{c}}\right|+{\text{SD}}_{\text{c}}*3,$$

where C indicates the currents recorded during dense noise stimulation. To quantify the postsynaptic currents, we calculated the amplitude and rise time of each detected wave event. The rise time was defined as the time required for the response to reach from 63% to 100% of the peak.

#### Analysis of Membrane Properties

To characterize the membrane properties of GCs, we recorded the membrane potential changes induced by current pulses (duration, 200 ms; amplitude, from −50 to 100 pA; increment, 10 pA). For hierarchical clustering (Figure 1F), we used three features (Supplementary Figure S1D): the resting membrane potential, the maximum instantaneous firing rate, and the input resistance. The resting membrane potential was calculated as an average potential 100 ms before application of current pulses. The maximum firing rate was calculated as an inverse of minimum inter-spike intervals during current pulses [Supplementary Figure S1D, (i)]. The input resistance was calculated as a slope of the current-voltage function below subthreshold membrane potentials [Supplementary Figure S1D, (ii)]. We performed a hierarchical clustering based on the correlation distance in the standardized feature space using *linkage* and *dendrogram* functions in MATLAB (Baden et al., 2016).

#### Morphological Analysis

Lucifer yellow was introduced through a recording patch pipette to visualize the cell morphology. Using ImageJ (NIH) and customized program in MATLAB, we quantified the dendritic field, shape, and dendritic branches of each GC (Figure 1 and Supplementary Figure S1E). The dendritic tips of a stained GC were fitted to a polygon, and then its major axis and the aspect ratio of major to minor axis were used as the measure of the length and the shape, respectively. The number of branches normalized by an area of the polygon was used as the measure of the dendritic branches.

### Quantification and Statistical Analysis

In Figures 2F–H, Kolmogorov-Smirnov test (KS test) was used. In Figure 2H, Hodges-Ajne test was used to evaluate the bias in distribution of the RF orientation. In Figures 3C,F, 4F, paired *t*-test was used. In Figure 5C, Mann-Whitney *U* test (MWU test) with Tukey’s *post hoc* test was used. All measures for population data were described as mean ± SD. Error bar indicates SD. ^∗∗∗^*p* < 0.001; ^∗∗^*p* < 0.01; ^∗^*p* < 0.05.

## Results

### Classification of Retinal Ganglion Cells Based on the Physiological and Anatomical Features

Applying a multi-electrode array to the goldfish isolated retina, we estimated the spatiotemporal receptive field (RF) profiles of retinal ganglion cells (GCs) by the reverse correlation method (Meister et al., 1994). The shape of the temporal RF is one of the criteria to define ganglion cell types functionally (DeVries and Baylor, 1997; Hilgen et al., 2017; Matsumoto and Tachibana, 2017). To dissect GC types, we performed feature detection using principal component analysis for the temporal RF (Matsumoto and Tachibana, 2017), and the resulting features were clustered into six GC groups based on k-means clustering (Figures 1A,B and Supplementary Figure S1B; Rousseeuw, 1987; Yuan and Yang, 2019). The differences among the GC groups were summarized by two temporal features: peak time (Fast/Medium/Slow; Figure 1C) and kinetics (transient/sustained; Figure 1D). Thus, individual GC groups were defined as Fast-transient (Ft, group 1), Fast-sustained (Fs, group 2), Medium-transient (Mt, group 3), Medium-sustained (Ms, group 4), Slow-transient (St, group 5), and Slow-sustained (Ss, group 6) GCs (Figure 1B and Supplementary Figure S1C).

We then assessed whether each GC group could share common physiological and anatomical features. We performed whole-cell recordings from randomly selected GCs and dye loading through the recording pipette to visualize the cell morphology (Supplementary Figures S1D–F). Based on three physiological features which represent the membrane properties (Figure 1E, i–iii) and two morphological features (Figure 1E, iv, v), a hierarchical clustering (Martínez et al., 2017; Gouwens et al., 2019) revealed that GCs were branched into six types (Figure 1F, type I to VI). Intriguingly, each type defined by physiological and anatomical features was populated dominantly by a GC group defined by the filtering property (Figures 1G,H). These results highlight the correlation between the filtering property and the physiological and anatomical features to characterize GCs.

### Global Jitter Motion Changes Spatiotemporal Receptive Field Profiles of a Specific Group of Retinal Ganglion Cells

To explore how the RF properties of GCs are affected by fixational eye movements, dense noise stimulus was presented on a large background (BG; 4 × 4 mm on the retina, visual angle >∼67°; Figure 2A; Macy and Easter, 1981). We used three kinds of BG patterns: a uniformly dark pattern (“Dark BG”), a static random-dot pattern (“Static BG,” a Gaussian-filtered random-dot pattern; mean luminance, 6.67 cd/m^2^), and a jittered random-dot pattern (“Jittered BG,” the Gaussian-filtered random-dot pattern jittered randomly) (Matsumoto and Tachibana, 2017). The jitter motion was a horizontally biased random walk (probability of horizontal shift/vertical shift = 2; each shift, 4 μm/50 ms; Figures 2B,C and Supplementary Figure S2C) that simulated the goldfish fixational eye movements (Easter et al., 1974; Mensh et al., 2004). Both the Static and Jittered BGs covered the area far away from the RFs of recorded GCs (Supplementary Figure S2A). Based on the responses to dense noise stimulus, we estimated the spatiotemporal RF of GCs under different BG conditions: RF_Dark BG_ (gray), RF_Static BG_ (black), and RF_Jittered BG_ (blue).

We found that the Jittered BG altered the spatiotemporal RF profile of Ft GCs (Figure 2D). The size of RF_Jittered BG_ (major axis of the RF; 161.9 ± 13.2 μm, 21 Ft GCs) was larger than that of either RF_Dark BG_ (112.5 ± 13.7 μm, *p* = 0.001; paired *t*-test) or RF_Static BG_ (115.6 ± 22.9 μm, *p* = 0.002). Expansion index, the ratio of the RF size under different BG conditions, was large only under the Jittered BG condition (Jittered BG vs. Dark BG, *p* = 8.91 × 10^–9^; Static BG vs. Dark BG, *p* = 0.38; KS test, 21 Ft GCs; Figure 2F, red). Other GC groups did not show prominent changes (Figures 2E,F, gray).

The temporal profile of RF_Jittered BG_ in Ft GCs showed faster peak time and smaller peak amplitude than those of RF_Dark BG_ and RF_Static BG_ (Figures 2D,G), indicating that the Jittered BG sensitized firing and input integration kinetics. These alterations of the temporal RF profile were specific to Ft GCs (peak time, Jittered BG vs. Dark BG, *p* = 2.35 × 10^–9^, Static BG vs. Dark BG, *p* = 0.097; peak amplitude, Jittered BG vs. Dark BG, *p* = 9.03 × 10^–9^, Static BG vs. Dark BG, *p* = 0.084; KS test, 21 Ft GCs; Figure 2G, red). The Jittered BG did not affect the temporal RF profile of other GC groups (Figures 2E,G, gray), indicating that the RF alterations are not ascribed to light adaptation to the random-dot background.

Intriguingly, RF_Jittered BG_ of Ft GCs was oriented along the retinal rostral-caudal (horizontal) axis (*p* = 0.019, Hodges-Ajne test, 21 Ft GCs; Figure 2H, blue), indicating that the RF expansion is biased along the retinal horizontal axis but not along the dorsal-ventral (vertical) axis (Figure 2H, bottom, red arrows). Since RF_Dark BG_ and RF_Static BG_ did not show orientation bias (RF_Dark BG_, *p* = 0.7763; RF_Static BG_, *p* = 0.7763; Hodges-Ajne test, 21 Ft GCs), the horizontal bias is not intrinsic to Ft GCs. Other GC groups showed no significant orientation bias under three BG conditions. It should be noted that the non-coherent (flickering) random noise BG did not alter the spatial RF profile of Ft GCs (RF size, Dark BG, 125.6 ± 21.1 μm; Flickering random noise BG, 121.9 ± 17.6 μm, *p* = 0.31; paired *t*-test, 17 Ft GCs; Supplementary Figure S2B). Therefore, global jitter motion is essential for the RF alterations in Ft GCs.

### Synaptic Currents Evoked by the Dense Noise Stimulus Under the Jittered BG Condition in Ft GCs

Effects of the global jitter motion on the spatiotemporal RF profiles were prominent in Ft GCs. To elucidate the underlying mechanisms, we recorded postsynaptic currents from Ft GCs in the whole-cell voltage-clamp configuration (Figure 3). Ft GCs were identified based on the temporal RF profile estimated by firing to dense noise stimulus in the cell-attached configuration. We found that excitatory postsynaptic currents (EPSCs) under the Jittered BG condition were larger in amplitude (Jittered BG, –110.2 ± 56.8 pA; Dark BG, –42.8 ± 33.9 pA; *p* = 0.001, paired *t*-test, 5 Ft GCs), and faster in kinetics (Jittered BG, –110.2 ± 56.8 pA; Dark BG, –42.8 ± 33.9 pA; *p* = 3.85 × 10^–5^, paired *t*-test, 5 Ft GCs) than those under the Dark BG condition (Figures 3B,C). EPSCs under the Static BG condition were not significantly different in both amplitude and kinetics from those under the Dark BG condition (Static BG, –45.7 ± 20.9 pA, 10.6 ± 2.5 ms, *p*s > 0.3; paired *t*-test, 5 Ft GCs; Supplementary Figure S3), indicating that emergence of large and fast EPSCs is not ascribed to adaptation to mean contrast increment (Dark BG vs. Static BG) but to alteration induced by the global jitter motion (Static BG vs. Jittered BG).

In contrast, inhibitory postsynaptic currents (IPSCs; Figure 3D) were not affected by the Jittered BG (amplitude, Jittered BG, 99.1 ± 64.5 pA; Dark BG, 102.6 ± 66.9 pA; kinetics, Jittered BG, 28.1 ± 15.5 ms, Dark BG 27.2 ± 16.7 ms, *p*s > 0.3; paired *t*-test, 5 Ft GCs; Figures 3E,F). Therefore, it is likely that the RF alterations under the Jittered BG condition were induced not by feedforward inhibitory synaptic inputs from amacrine cells (ACs) to Ft GCs but by excitatory synaptic inputs from bipolar cells (BCs) to Ft GCs.

### Alterations of the Spatial Distribution of Excitatory Inputs to Ft GCs by the Jittered BG

To examine the spatial RF profile under the Jittered BG condition in detail, we mapped the distribution of EPSCs evoked by local light stimulation (Spatial EPSC map; Figure 4). A whole-cell voltage-clamped Ft GC was stimulated by a small flash (Figure 4A; size, 100 × 100 μm; duration, 50 ms; inter-flash interval, 1 s), which was presented randomly at various locations on the Dark BG (Figure 4B) or on the Jittered BG (Figure 4C). We found that the EPSC map under the Jittered BG condition was spatially expanded along the retinal horizontal axis (Figures 4C–E), corresponding to the horizontally elongated RF_Jittered BG_ (Figure 2D).

Interestingly, we found that the properties of evoked EPSCs were not homogeneous across the RF under the Jittered BG condition: small and slow inputs at the center region (Figure 4D [2] and Figure 4F; 17.8 ± 9.87 pA, 9.54 ± 1.54 ms); large and fast inputs at the horizontally-expanded region (Figure 4D [1] and Figure 4F; 43.7 ± 19.39 pA, 7.54 ± 1.53 ms). It is likely that the emergence of large and fast EPSCs mediates the horizontally elongated RF of Ft GCs under the Jittered BG condition.

### Contribution of GABAergic and Electrical Pathways to RF Elongation in Ft GCs

In the goldfish retina both GABAergic inhibition from AC to Mb1 (ON) BC terminal (Tachibana and Kaneko, 1988) and electrical coupling through connexin 36 (Cx36) between Mb1 BC dendrites contribute to global information processing (Arai et al., 2010; Tanaka and Tachibana, 2013; Matsumoto and Tachibana, 2017). It is possible that the horizontal RF elongation in Ft GCs may be attributable to deactivation of lateral inhibition mediated by ACs and/or activation of electrically coupled excitatory network (Bloomfield and Völgyi, 2009; Werblin, 2011).

Using a multi-electrode array, we examined the effects of pharmacological blockers on the properties of RF_Jittered BG_ (Figure 5). Application of a GABA receptor blocker picrotoxin (PTX, 100 μM) increased the RF_Dark BG_ size (horizontal length, 142.7 ± 12.4 μm in Control, 162.3 ± 18.1 μm in PTX, *p* = 0.03; vertical length, 133.5 ± 13.9 μm in Control, 158.6 ± 14.8 μm in PTX, *p* = 0.01; Mann-Whitney *U* (MWU) test, 7 Ft GCs; Figures 5B,C). Even in the presence of PTX, the Jittered BG increased further the RF size (RF_Jittered BG_, horizontal length 194.5 ± 17.9 μm, *p* = 0.007; vertical length 208.2 ± 27.3 μm, *p* = 0.0012; MWU test, 7 Ft GCs, Figure 5C), although the spatial bias disappeared (Figure 5D). These results suggest that two different GABAergic mechanisms may mediate the surround inhibition (Werblin, 2011): the local inhibition which works under the dark BG condition, and the global inhibition which contributes to emphasizing the horizontal bias of the RF under the Jittered BG condition.

In the presence of gap junction blocker mefloquine (MFQ, 10 μM; Cruikshank et al., 2004), the Jittered BG did not increase the horizontal length of the RF_Jittered BG_ (horizontal length, Dark BG, 140.7 ± 16.1 μm; Jittered BG, 132.7 ± 14.8 μm, *p* = 0.32; MWU test, 7 Ft GCs; Figures 5B,C), but decreased the vertical length of the RF_Jittered BG_ significantly (vertical length, 130.3 ± 15.9 μm under the Dark BG, 107.3 ± 8.3 μm under the Jittered BG, *p* = 0.0175; MWU test, 7 Ft GCs; Figure 5C). These results suggest that electrical coupling may mediate the spatial expansion of the RF_Jittered BG_. Indeed, spatial bias index (see section “Method Details”) indicates that the horizontal bias of the RF_Jittered BG_ was still maintained in the presence of MFQ (Figure 5D, green), whereas the horizontal bias was not observed in the presence of PTX (Figure 5D, red).

Therefore, the potential mechanism for the RF alterations may be the following: the Jittered BG expands the RF in all directions by electrical coupling, perhaps between Mb1 BCs (Arai et al., 2010); and the dorsal and ventral sides of the expanded RF are suppressed by activated GABAergic lateral inhibition (Tanaka and Tachibana, 2013), resulting in the horizontally elongated RF (Figure 5E). Lateral inhibition may be ascribed not to the feedforward input from AC to Ft GC but to the feedback input from AC to Mb1 BC terminal (Tanaka and Tachibana, 2013) because IPSCs in Ft GCs were not altered by the Jittered BG (Figures 3E,F).

## Discussion

In our previous study, we showed that firing properties of specific GCs in the goldfish retina were modulated by a rapid shift of a target following a period of jitter motion of a global random-dot background (Matsumoto and Tachibana, 2017). Here, we examined how global jitter motion alters the RF properties of Ft GCs and what mechanisms underlies the RF alteration. We found that global jitter motion induced the RF elongation along the retinal horizontal (caudal-rostral) axis (Figure 2) in Ft GCs (Figure 1). At the elongated region, EPSCs with large amplitude and fast kinetics were dominated (Figures 3, 4). Pharmacological experiments revealed that both GABAergic lateral inhibition and electrical coupling contributed to the horizontal RF elongation (Figure 5).

### Synaptic Modulation Mediated by Electrical and GABAergic Pathways

Our results indicate that the RF alteration in Ft GCs is mediated by lateral interaction pathways: electrical and GABAergic pathways (Figure 5). In the goldfish retina, excitation of an Mb1 BC spreads to neighboring Mb1 BCs through gap junction between their dendrites (Arai et al., 2010). Such lateral spread of excitation could expand the RF. It is possible that the synchronous transient glutamate release from electrically coupled BCs may evoke EPSCs with large amplitude and fast kinetics. It is conceivable that Ft GCs may receive excitatory inputs from a population of BCs under the Dark BG (“conventional BCs” in Figure 5E), and large and fast excitatory inputs from another population of electrically coupled BCs which are additionally activated by the Jittered BG (“Electrically coupled BCs” in Figure 5E).

In the goldfish retina, global stimulation activates the electrically coupled Mb1 BC network, which in turn activates wide-field ACs and/or electrically coupled ACs (Tanaka and Tachibana, 2013). Blockade of GABA receptors under the Dark BG condition increased the size of RF in all directions (Figure 5C), indicating that the local GABAergic inhibition was not biased spatially (Figure 5E). However, blockade of GABA receptors under the Jittered BG condition impaired the horizontal bias (Figure 5D). These observations indicate that the global inhibitory mechanism could affect the electrically coupled BCs at the dorsal and vertical side of the expanded RF (“Global GABAergic inhibition,” Figure 5E).

On the other hand, the horizontal bias remained even after blockade of electrical coupling (Figure 5D). This result indicates that the inputs from electrically coupled BCs may not be the unique resource to drive the global inhibitory mechanism. It is possible that the continuous jitter motion may prevent the BCs from adaptation, resulting in augmentation of the synaptic inputs to the wide-field ACs. Another possible resource may be the ACs electrically coupled through non-Cx36 (Mark et al., 1988; Völgyi et al., 2013), which are also activated by the Jittered BG. This mechanism could explain the observed horizontal bias in the presence of a gap junction blocker. Since AC circuits in the goldfish retina are not yet fully understood, further studies are needed to clarify the circuit mechanism.

### Diverse Features of Fixational Eye Movements

In this study we used randomly jittering motion to mimic the retinal image during fixation, which was similar to the random walk used in the previous studies (Ölveczky et al., 2003; Baccus et al., 2008; Segal et al., 2015). The stimulus was summarized by oscillatory image motion with small amplitude, which approximates to an animal’s tremor (e.g., archer fish, 12 μm in mean amplitude, 5 Hz in frequency, Segev et al., 2007). Nevertheless, the features of fixational eye movements are more complicated in amplitude, speed, and frequency: drift, slow motion occurring together with tremor; microsaccedes, small but rapid and involuntary shifts of eyes (Martinez-Conde et al., 2004; Rucci and Poletti, 2015). Global jitter motion used in this study may be similar to tremor and drift in periodicity and slow frequency components (0.5–8 Hz; Supplementary Figure S2C). On the other hand, our stimulus did not include the small and rapid displacements corresponding to microsaccades. Furthermore, it is not yet clear how three components of fixational eye movements interact with one another and affect the retinal circuit. It is likely that that the RF alterations shown in this study may be a part of the effects induced by fixational eye movements. Further studies are required to dissect the relationship between the diverse features of fixational eye movements and the retinal information processing.

### Functional Relevance of RF Elongation

Goldfish makes horizontally biased saccades spontaneously (Salas et al., 1997). Thus, the horizontal elongation of RF in Ft GCs during global jitter motion seems to be advantageous to responding quickly to a target in a succeeding rapid gaze shift (saccade). Indeed, we have shown that horizontal, but not vertical, rapid shift of a target following jitter motion evoked firing in Ft GCs before arrival of the target to the RF estimated under the Dark BG condition (Matsumoto and Tachibana, 2017). In some visual neurons in the brain, their RFs spatially shift or expand toward the future position of eyes before saccade initiation (frontal eye field; Umeno and Goldberg, 1997, prefrontal cortex; Zirnsak et al., 2014). These dynamic RF changes in visual neurons may contribute to fast and efficient processing of visual information during repetitive sequence of eye movements (Hosoya et al., 2005; Gollisch and Meister, 2008; Wurtz, 2008; Zirnsak et al., 2014). It is possible that, in natural environment, the RF profiles of specific GCs are tuned to global image motion induced by eye movements to facilitate processing in the visual system.

## Data Availability

The data and codes that used in this research are available at the corresponding author, MT (mstchbn@fc.ritsumei.ac.jp) upon reasonable requests.

## Ethics Statement

The animal study was reviewed and approved by Committee of Animal Experiments in The University of Tokyo.

## Author Contributions

Both authors designed the study, performed all experiments, and wrote the manuscript. AM performed the data analysis.

## Conflict of Interest Statement

The authors declare that the research was conducted in the absence of any commercial or financial relationships that could be construed as a potential conflict of interest.
