# Corrigendum: Global Jitter Motion of the Retinal Image Dynamically Alters the Receptive Field Properties of Retinal Ganglion Cells

**DOI:** 10.3389/fnins.2020.00234

**Published:** 2020-03-24

**Authors:** Akihiro Matsumoto, Masao Tachibana

**Affiliations:** ^1^Department of Psychology, Graduate School of Humanities and Sociology, The University of Tokyo, Tokyo, Japan; ^2^Ritsumeikan Global Innovation Research Organization (R-GIRO), Ritsumeikan University, Kusatsu, Japan; ^3^Danish Research Institute of Translational Neuroscience (DANDRITE), Department of Biomedicine, Aarhus University, Aarhus, Denmark; ^4^Research Organization of Science and Technology, Ritsumeikan University, Kusatsu, Japan

**Keywords:** retina, retinal ganglion cells, receptive field, eye movements, gap junctions

In the original article, an author's name was incorrectly spelled as **Akihiro Matsutmoto**. The correct spelling is **Akihiro Matsumoto**.

The authors apologize for this error and state that this does not change the scientific conclusions of the article in any way. The original article has been updated.

