# Supplementary material for: Global Jitter Motion of the Retinal Image Dynamically Alters the Receptive Field Properties of Retinal Ganglion Cells
Source: Front Neurosci. 2019 Sep 13;13:979. doi: 10.3389/fnins.2019.00979 (PMC6753181; doi:10.3389/fnins.2019.00979)
Supplement: Supplementary file 1 [file Presentation_1.pdf]

Supplementary Material

Global jitter motion of the retinal image dynamically alters the receptive field properties of retinal ganglion cells.

Akihiro Matsumoto, Masao Tachibana

1 Supplementary Figures and Tables

Supplementary Figure 1, Related to Figure 1 and 2.

Supplementary Figure 2, Related to Figure 2.

Supplementary Figure 3, Related to Figure 3.

1.1 Supplementary Figures

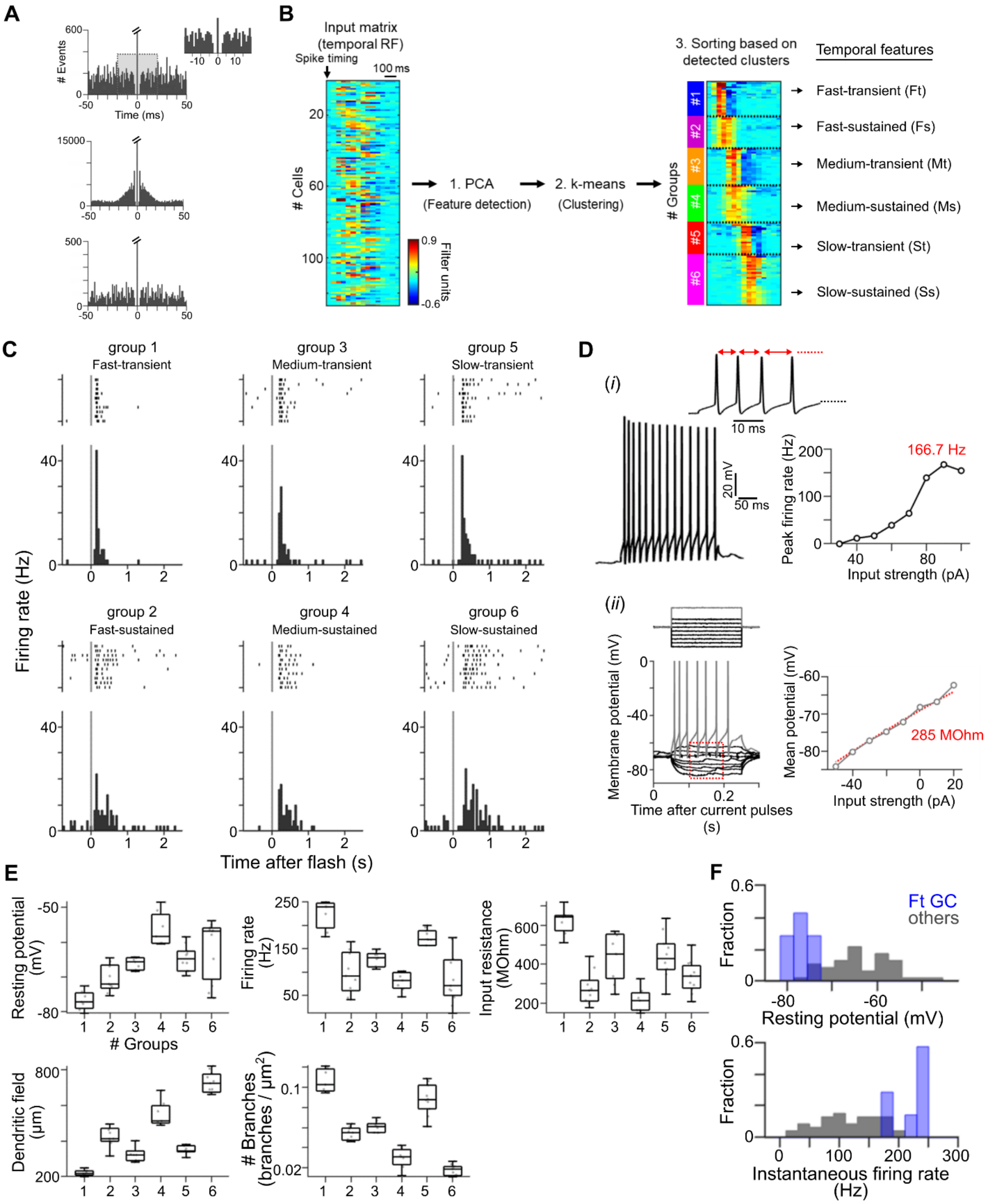

Supplementary Figures S1. Physiological and morphological classification of goldfish retinal ganglion cells. (A) Auto-correlograms of spike trains from isolated three units. Inset, a magnification in the range of -20 to +20 ms time delays (gray in top). We used units which showed the refractory

period of spikes for further analysis. **(B)** GC group classification based on the temporal profile of the receptive field (temporal RF). The temporal RF of each GC was estimated from the multi-electrode recordings, and input matrix was created (Left, heat map). Then, using principal component analysis (PCA) and k-means clustering, GCs were sorted into 6 groups (Right, heat map). **(C)** Examples of light-evoked responses in each GC group. Black dots, raster plots of 10 trials. Black bars, peri-stimulus time histograms (50 ms bin width). Gray line, onset of the light stimulus (duration, 1 s; contrast, 100 %). **(D)** Definition of the instantaneous firing rate (***i***) and the input resistance (***ii***). Inter-spike interval of the firing evoked by current pulses (−50 to 100 pA, 10 pA increment) was measured (red arrows), and an inverse of its minimum value was defined as the peak firing rate (***i***). The input resistance was calculated as a slope of the current-voltage function below subthreshold membrane potentials (***ii***). **(E)** Summaries for five features: the resting membrane potential, the peak firing rate, the input resistance, the length of dendritic field, and the branch number of dendrites. These features were used for a hierarchical clustering (Figure 1). 7 group1 (Ft), 8 group2 (Fs), 7 group3 (Mt), 9 group4 (Ms), 9 group5 (St), 12 group6 (Ss) GCs. **(F)** Fraction of the resting potential (upper) and instantaneous firing rate (lower) distributions for Ft GCs (blue, 7 cells from group1) and other GCs (gray, 45 cells). Ft GCs were characterized by a more negative resting membrane potential and a higher instantaneous firing rate of responses (–76.5 ± 3.2 mV, 222.9 ± 90.7 Hz, 7 Ft GCs) than other GCs (–64.5 ± 7.4 mV, 125.3 ± 67.3 Hz, 45 other GCs).

1.2 Supplementary Figures

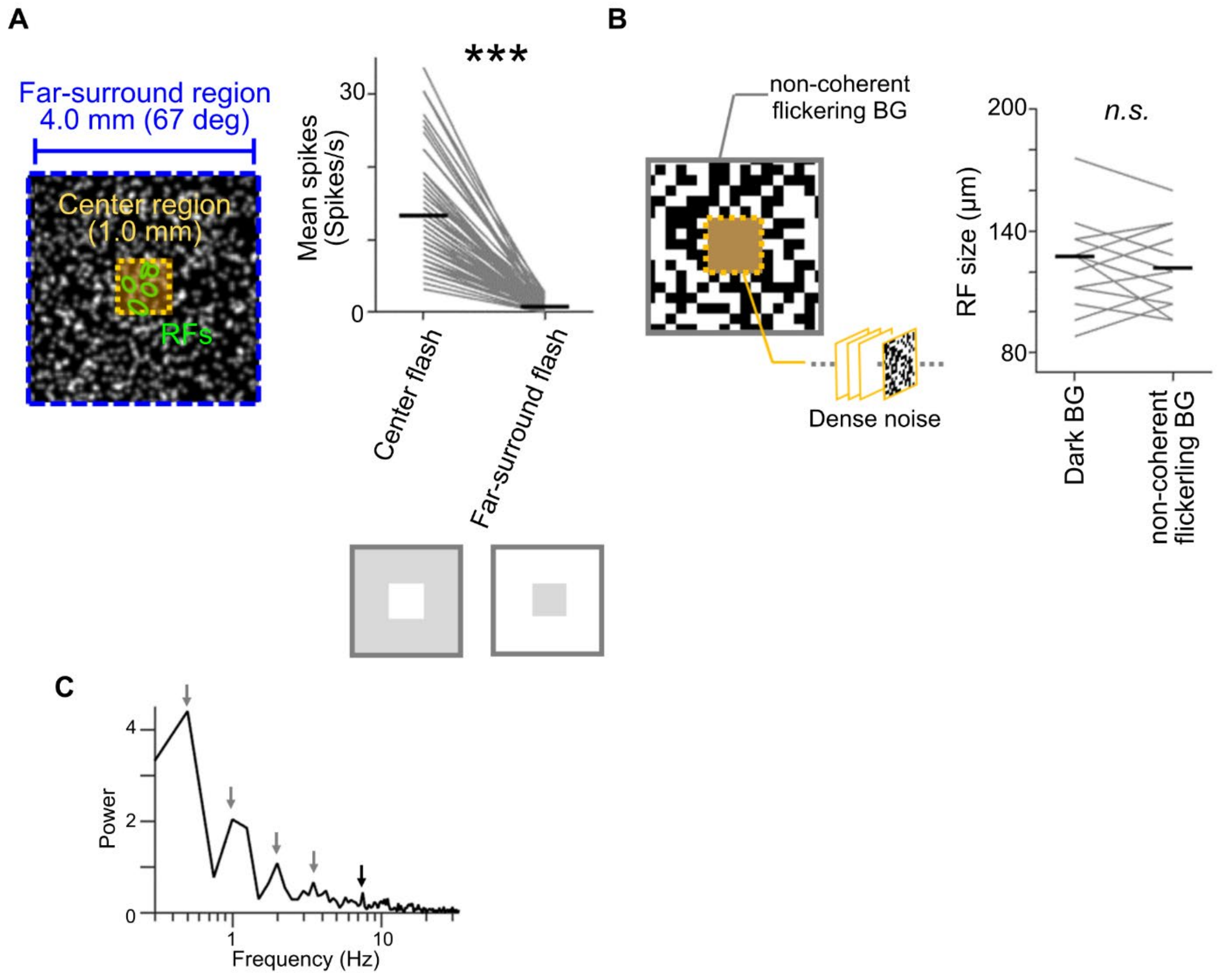

Supplementary Figures S2. Stimulation at the far-surround region. (A) Configuration of the jittered global background (Left). GCs on the multi-electrode array responded to a center flash but not to a far-surround flash (Right). 72 GCs, 5 retinas. ***, $p < .001$, paired *t*-test. **(B)** Effects of the non-coherent flickering BG on the RF of Ft GCs. The RF was estimated by the dense noise stimulus at the center region while a randomly flickering checkerboard BG was presented on the far-surround region (Left). The RF size under the non-coherent flickering BG condition was not significantly different from that under the Dark BG condition (Right). 17 Ft GCs, 3 retinas. *n.s.*, paired *t*-test. **(C)** Fast Fourier Transform to estimate the frequency components of the jitter motion trajectory (**Figure 2B**). Arrows, subpeaks shown in the spectrum.

1.3 Supplementary Figures

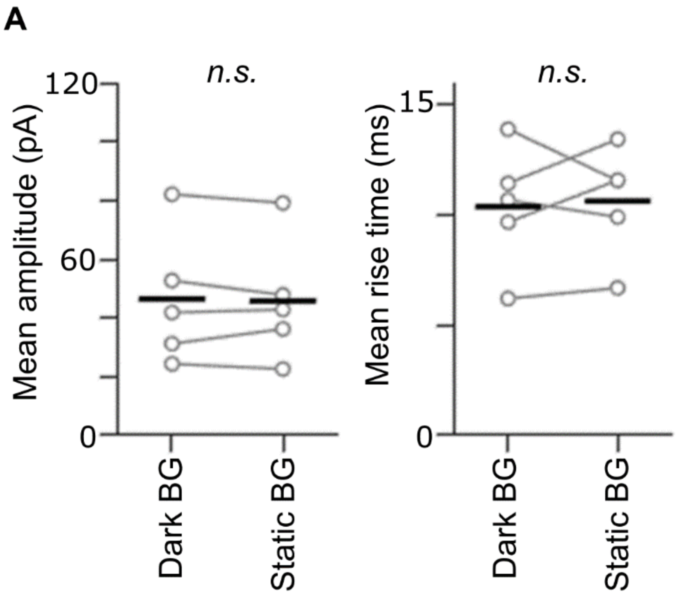

Supplementary Figures S3. EPSCs evoked by dense noise stimulation under the static BG condition. (A) Mean amplitude (Left) and rise time (Right) of EPSCs recorded from whole-cell voltage-clamped Ft GCs. Holding potential was −60 mV. Properties of EPSCs evoked under the Static BG condition was not significantly different from those under the Dark BG condition. 5 Ft GCs, 5 retinas. *n.s.*, paired *t*-test.
